# The Spread of Plague by Fleas

**Published:** 1903-12-05

**Authors:** 


### THE SPREAD OF PLAGUE BY FLEAS.

In an interesting communication on the subject Dr. Elkington[1] gives a summary of the evidence which can be produced in favour of the theory that fleas play an important part in the convection of plague. In 1897 Dr. Simond succeeded in conveying plague to a mouse by injecting an emulsion of fleas which had been fed on a plague-infected animal. His observations, which were communicated to the annals of the Pasteur Institute, did not attract very much attention at the time. The final proof, however, that other diseases were dependent for their spread upon suctorial insects, *e.g.*, malaria and mosquitos, turned the notice of investigators once more towards the possibilities of plague convection by fleas.

Before summarising the evidence against the flea, Dr. Elkington premises that there are other sources of contagion. In rats, at any rate, the disease may be induced by feeding upon the fresh organs of animals which have died of the plague, and in the human being there is no reason to connect plague pneumonia with the bites of infected fleas In favour of the theory that these insects can and do convey the disease are the following points. In the first place bubonic plague is essentially a place disease. The Indian Plague Commission reported that "the universal experience of plague in India proves . . . that houses into which the infection of plague has been imported, whether by men or rats, are infective, this infectivity being so marked that many of the officers who have had most experience of the disease have come to the conclusion that the principal source of infection is . . . to be found in the houses into which the infection of plague has been introduced." Much work has been done to discover, if possible, the power of survival of the plague bacillus outside the animal body. The earthen floors of plague-stricken native houses should be especially liable to contain the organism were it capable of growing thereon; yet out of all the experiments which have been done in India, with a view to detecting it, the plague bacillus has never been found in such floors. It has, furthermore, been proved that even when introduced in great quantities, under experimental conditions, into soil, the organism is only recoverable for a few days afterwards. Another curious point about this house infection is that it is mostly confined to particular kinds of houses, namely, the dark, dirty, squalid, vermin-haunted abodes of the poorest classes.

Again, the infectivity of clothing is another marked feature of plague, the incidence of the disease upon native washermen being very marked. That this infectivity of clothing is not due to plague bacilli lying free in the clothes is indicated by the fact that numerous bacteriological examinations of the clothing of persons who had plague or who had died of plague failed to show any trace of the organism. Dr. Elkington believes that the infectivity of clothes is probably due to fleas which they harbour. One more important point bearing on the matter is that in the very great majority of septicæmic and bubonic cases the entry of the plague bacillus is effected through the skin — a point upon which authorities agree.

The religious sect known as the Jains carry out the law of protection of all forms of animal life to its extremist limits, consequently their homes are swarming with vermin. As a class the Jains show an extraordinary liability to plague and are especially smitten in every outbreak. Their commonest suctorial companions are fleas, bugs, and flies. It has been shown by Dr. Nuttall that bugs do not transmit plague among animals, and ordinary flies die rapidly when fed on plague-infected material. Fleas, as will be shown later, do not die when fed upon plague-infected animals.

It has been noticed in India that, while the coolies of disinfecting gangs show no marked mortality so long as they sleep in uninfected localities, so soon as they rest in an infected locality they are especially liable to plague. This seems to indicate that the agent of convection of plague is most active at night or while the subject is resting. Now the flea is an animal of nocturnal habits. It has a strong objection to strong light, and its domicile is in the clothing and, for the most part, the bedding of its host. It is generally conceded that rats play an important part in plague epidemics, and here again Dr. Elkington brings forward evidence to show that the flea is the actual medium by which infection is conveyed from diseased rats to man. While the different species of fleas show a preference for the particular animal with which they are usually associated they are catholic in their tastes when hungry, and, under experimental conditions, rat fleas will bite human beings, and human fleas will feed upon rats. Further experiments have proved that fleas when fed upon infected rats, and also when fed upon human beings who are suffering from plague, are capable of transmitting the disease to healthy rats simply by being allowed to bite them. Lastly by feeding fleas on infected animals and then killing them and cutting them into sections, the bacilli can be found in large numbers in the fleas' gizzards even up to 84 hours after the meal, and it is thought likely that they may remain there alive for a much longer period. When a flea "bites" it injects a secretion, and it is highly probable that in the case of an infected flea some of the bacilli may be injected with this secretion.

The above facts form very strong evidence, although in the absence of human experiments absolute proof may yet be wanting, in favour of the proposition that fleas are very important agents, if not the most important, in the spread of bubonic plague.

[1] Australasian Medical Gazette.

